# Neuro-optometric treatment for visual snow syndrome: recent advances

**DOI:** 10.2217/cnc-2023-0006

**Published:** 2023-05-30

**Authors:** Kenneth J Ciuffreda, Barry Tannen, Daniella Rutner, MH Esther Han

**Affiliations:** 1 SUNY/College of Optometry, Vision Rehabilitation Service, Visual Snow Group, New York, NY 10036, USA

**Keywords:** chromatic filters, concussion, neuro-optometry, oculomotor therapy, visual snow, visual snow syndrome

The condition of visual snow syndrome (VSS) is characterized by the presence of a pixelated overlay of ‘visual snow’ (VS) throughout the visual field, that is dynamic visual noise [1]. It is found in approximately 2% of the general population [1]. In addition, a constellation of related sensory, motor and perceptual abnormalities is present (e.g., palinopsia, photosensitivity and tinnitus) [1]. Its primary etiology is concussion [1]. While VSS has received considerable attention regarding its defining characteristics and diagnostic criteria [2,3], the area of treatment has been relatively neglected [4], and it has not been promising thus far. For example, medications have had minimal success (∼20%), and in many cases (up to 35%) have exacerbated the primary symptom of visual snow [4]. In this editorial update [5], recent advances in the neuro-optometric treatment approach to VSS will be considered in patients with concussion and other neurological events.

The first report to provide evidence for chromatic filters to reduce VS was by Lauschke *et al.* in 2016 [6]. Twelve individuals with VSS were assessed with the intuitive colorimeter (IC) (Cerium Visual Technologies, Tenterden, Kent, UK) for a precision chromatic tint to reduce their VS. Eleven of the 12 (92%) selected a specific precision tint that reduced the VS, with subsequent testing producing the same tint result. This positive finding suggested that the prescription of chromatic tints might be a fruitful treatment direction to pursue in these patients.

Over the past 6 years, the SUNY Visual Snow Group has published several papers that have confirmed and extended considerably the aforementioned findings [1,5,7–12]. Over 100 patients diagnosed with VSS have been carefully assessed using the IC and/or specially designed test procedures/protocols by us. These studies were performed independently in two clinical environments, one academic and one private practice. The overall, major finding was that, on average, 80–90% preferred a specific, precision, chromatic tint [9,12] for either general wear or for a specific usage (e.g., iPhone and computer tasks) [8], similar to the percentage reported by Lauschke *et al.* [6]. Some of the most commonly prescribed tints (and their average transmission) were (Brain Power, FL, USA): FL-41 (25%, 50%), BPI-Omega (50%) and BPI-Mu (73%). These chromatic tints typically reduced light transmission over the shorter wavelengths of the visible spectrum (e.g., ‘blue’) [12].

In addition, the chromatic filters reduced the related abnormal phenomena of palinopsia and photosensitivity [9], on average by 50%, with a range from 10 to 100% in perceived reduction. To reduce further the palinopsia, systematic saccadic tracking over a range of amplitudes (5–15°) and in multiple directions (i.e., horizontal, vertical and oblique) was instituted as part of the weekly general oculomotor therapy program, for up to 16 weeks [9], with a high level of success (>90%). We hypothesized that the tracking re-established more normal levels of saccadic suppression [13], with this abnormal perception of one’s saccadic trajectory/afterimage due to a neurological disinhibition process [7,9].

A recent and important new finding in this population has been the very high prevalence of oculomotor dysfunctions based on conventional clinical criteria (e.g., convergence insufficiency, saccadic dysmetria) [9,12]. It was found to be approximately 60% in the VSS cohorts tested, which is more than twice that found in the non-VSS general population (up to 25%) [14]. This finding has been substantiated in part by recent laboratory-based, saccadic eye movement studies [15–17]. These common, clinically-based, oculomotor deficits were remediated in >90% of the patients [9] by conventional, oculomotor therapy [18].

Recently two therapeutic protocols for VSS have been published, which should provide for more uniform evaluation and treatment guidelines, as well as improved patient care, especially when used in combination. One is primarily oculomotor/motor-based therapy [19], whereas the other is more comprehensive (i.e., tints and oculomotor-based therapy) and includes all of the aforementioned visual deficits found and currently treatable in the VSS population [11].

The presence of visual snow and its related visual disturbances can have adverse effects on many common activities of daily living [8,9]. This might include: reading where the frequently occurring palinopsia is present with each successive saccadic-based, reading eye movement; thus the individual perceives the newly-fixated word with the afterimage of the previously fixated word superimposed, at times with trailing and when combined perceptually with the ever-present visual snow, creates a very disturbing and distracting scenario; and during computer usage where the overlay of the visual snow is present in the foreground and the important text is in the background, which creates two conflicting and disturbing visual planes likely to cause attentional disruptions and confusion; a third one is driving, which has recently been highlighted by us [10]. During driving, several of the visual snow syndromes, visual-perceptual aspects can have an adverse effect, with potential safety consequences. These include the visual snow, palinopsia, photosensitivity, enhanced entoptic imagery and photopsia. For example, the presence of visual snow, especially when combined with any entoptic imagery, would impair the accuracy of ocular accommodation/eye focusing of the pre-presbyopic driver, thus adding a small amount of blur (∼0.6 D) to the aforementioned disturbing visual snow and entoptic aspects. This would be especially true for night-time driving, where the driving visual context is already degraded due to the relatively low, ambient illumination and degraded vertical spatial frames-of-reference (e.g., houses, telephone poles, trees). In addition, the presence of two apparent perceptual depth planes, as mentioned earlier, can impact negatively on one’s distance judgement (e.g., one’s estimate of the physical spacing of two cars on the roadway), as well a cause a visual disturbance that could impair visual attention during the complex driving task. In another scenario, such as daytime driving on a sunny day, the individual’s photosensitivity could have a distracting visual and attentional element. All of the above have potential safety ramifications of a visual-attentional and visual-perceptual nature. Fortunately, there are simple remedies to reduce each of the above problems, as described earlier. These include chromatic tints for the visual snow, palinopsia, photopsia and enhanced entoptic imagery, and oculomotor-based saccadic tracking techniques to reduce the palinopsia and in turn decrease the potential safety concerns [10].

These recent neuro-optometric, therapeutic advances are promising. However, investigations into the sensory, motor, and perceptual dysfunctions and their clinical ramifications remains in its infancy. There are several possible future research directions. First, with respect to chromatic tints, there is a need for testing larger groups of individuals, including a randomized clinical trial to determine therapeutic efficacy/optimality, and furthermore to assess for a possible placebo component to the overall positive effects [12]. Second, additional psychophysical testing is warranted, for example to determine the underlying chromatic mechanisms/adaptative processes, and their possible interaction with the precision chromatic tint, especially over time [20]. Third, laboratory studies for the eye movement dysfunctions found clinically should be documented using an objective, quantitative approach [13]. At last, and related to all of the above, brain imaging studies are needed to determine the underlying neural mechanisms and brain sites to better understand this enigmatic condition, and thus provide more targeted and efficacious therapeutic interventions in patients with concussion and other neurological problems.

In conclusion, the condition of visual snow syndrome, which is found is found in approximately 2% of the general population, has received considerable attention over the past decade by both clinicians and researchers. Fortunately, two neuro-optometric rehabilitative approaches have benefitted most patients. This has included colored filters and eye movement procedures to reduce the perception of the visual snow and some of the other related visual disturbances, such as light sensitivity.
